# Impact of Built-up-Litter and Commercial Antimicrobials on *Salmonella* and *Campylobacter* Contamination of Broiler Carcasses Processed at a Pilot Mobile Poultry-Processing Unit

**DOI:** 10.3389/fvets.2017.00088

**Published:** 2017-06-09

**Authors:** KaWang Li, Lacey Lemonakis, Brian Glover, Joseph Moritz, Cangliang Shen

**Affiliations:** ^1^Davis College, Division of Animal and Nutritional Sciences, West Virginia University, Morgantown, WV, United States

**Keywords:** broiler carcasses, mobile poultry-processing unit, litter, antimicrobials, *Salmonella*, *Campylobacter*

## Abstract

The small-scale mobile poultry-processing unit (MPPU) produced raw poultry products are of particular food safety concern due to exemption of USDA poultry products inspection act. Limited studies reported the microbial quality and safety of MPPU-processed poultry carcasses. This study evaluated the *Salmonella* and *Campylobacter* prevalence in broiler ceca and on MPPU-processed carcasses and efficacy of commercial antimicrobials against *Campylobacter jejuni* on broilers. In study I, straight-run Hubbard × Cobb broilers (147) were reared for 38 days on clean-shavings (CS, 75) or built-up-litter (BUL, 72) and processed at an MPPU. Aerobic plate counts (APCs), coliforms, *Escherichia coli*, and yeast/molds (Y/M) of carcasses were analyzed on petrifilms. Ceca and carcass samples underwent microbial analyses for *Salmonella* and *Campylobacter* spp. using the modified USDA method and confirmed by API-20e test (*Salmonella*), latex agglutination immunoassay (*Campylobacter*), and Gram staining (*Campylobacter*). Quantitative polymerase chain reaction (CadF gene) identified the prevalence of *C. jejuni* and *Campylobacter coli* in ceca and on carcasses. In study II, fresh chilled broiler carcasses were spot inoculated with *C. jejuni* (4.5 log_10_ CFU/mL) and then undipped, or dipped into peroxyacetic acid (PAA) (1,000 ppm), lactic acid (5%), lactic and citric acid blend (2.5%), sodium hypochlorite (69 ppm), or a H_2_O_2_–PAA mix (SaniDate^®^ 5.0, 0.25%) for 30 s. Surviving *C. jejuni* was recovered onto Brucella agar. APCs, coliforms, and *E. coli* populations were similar (*P* > 0.05) on CS and BUL carcasses. Carcasses of broilers raised on BUL contained a greater (*P* < 0.05) Y/M population (2.2 log_10_ CFU/mL) than those reared on CS (1.8 log_10_ CFU/mL). *Salmonella* was not detected in any ceca samples, whereas 2.8% of the carcasses from BUL were present with *Salmonella*. Prevalence of *Campylobacter* spp., *C. jejuni* was lower (*P* < 0.05), and *C. coli* was similar (*P* > 0.05) in CS-treated ceca than BUL samples. Prevalence of *Campylobacter* spp., *C. jejuni*, and *C. coli* was not different (*P* > 0.05) on CS- and BUL-treated carcasses. All antimicrobials reduced *C. jejuni* by 1.2–2.0 log CFU/mL on carcasses compared with controls. Hence, raising broilers on CS and applying post-chilling antimicrobial treatment can reduce *Salmonella* and *Campylobacter* on MPPU-processed broiler carcasses.

## Introduction

Since July 2011, new performance standards have been established by the United States Department of Agriculture-Food Safety and Inspection Service (USDA-FSIS) in response to national baseline studies that required routine testing for *Salmonella* and *Campylobacter* in all processing plants. These new performance standards state that the percentage of *Salmonella*-positive samples must be <7.5% and *Campylobacter*-positive samples should be <10.4% (1). With the implementation of more rigorous standards for pathogen reduction by the USDA-FSIS, it is necessary for poultry processors to employ new or additional pre- and post-harvest interventions for effective control of *Salmonella* and *Campylobacter* throughout chicken processing.

Demand for locally grown products has increased due to consumer interest in sustainable agriculture and an expectation of improved flavor and nutrition. Interest in pastured poultry production and on-farm mobile slaughter of poultry has increased dramatically in the last 20 years. In the previous 5–10 years, some Mid-Atlanta states (Kentucky, Pennsylvania, Ohio, and Massachusetts) have offered mobile poultry-processing units (MPPUs) to small-scale farmers to facilitate production and processing of ≤1,000 broilers per year for local and intrastate, direct sale to consumers under the inspection exemption by the USDA-FSIS *Poultry Products Inspection Act*. According to the West Virginia Department of Agriculture (WVDA), no small-scale poultry-processing facilities (including MPPUs) exist at West Virginia (WV). The lack of small-scale poultry-slaughtering facilities limits small-scale poultry producers in WV to local/intrastate selling of ≤1,000 birds per year. Small-scale farmers who wish to slaughter and sell poultry products locally must have them slaughtered and processed in an out-of-state USDA-FSIS-inspected facility (2). To continue to grow small-scale local poultry industries at WV, the WVDA is planning to assist small-scale poultry processors to install MPPUs at state-wide areas (Personal communication with Mr. Jerry Ours, Poultry Program Coordinator of WVDA). Therefore, it is important to conduct research projects from pre-harvest to post-harvest process to identify food safety risks associated with locally produced broilers, to provide supporting documentation for implementation of an MPPU, to secure local production and distribution of safe poultry meat in WV, and eventually to decrease/eliminate health disparities through optimized local food systems in WV and the mid-Atlantic region.

From the pre-harvest prospective, locally small-scale poultry growers often reuse litter to rear consecutive broiler flocks. Litter is often reused for 1–2 years before full cleanout and replacing with new litter (3). Therefore, food safety concerns are raising about the reusing of litter especially for the challenge of control *Campylobacter* during poultry raising. There is limited research on the comparison of broilers reared on clean-shavings (SC) vs. built-up-litter (BUL) regarding the microbial quality and safety of broilers, including the colonization and contamination of *Salmonella* and *Campylobacter* spp. on broiler carcasses. The literature shows that the welfare, health, performance, and carcass quality of poultry are affected directly by litter quality (4). The samples utilized for the present study were collected from a small-scale broiler trial that compared the performance of industry-standard broilers reared on CS or BUL.

From the post-harvest processing prospect, the slaughter and carcass processing in MPPUs are carried out on a more manual basis instead of using industry-scale, large, automated commercial processing lines. Their products differ based on the variety of available equipment, producer resources, and facilities. This diversity, along with the absence of regulatory guidance, has failed to yield the data needed to validate the safety of raw chicken/broiler carcasses and chicken parts produced by MPPUs. The limited application of antimicrobial intervention plus a final ice water-chilling process without application of post-chilling decontamination treatments makes locally grown MPPU-processed poultry products more vulnerable to infection by *Salmonella* and *Campylobacter*. Lactic acid (LA), peroxyacetic acid (PAA), sodium hypochlorite (SH), and a blend of lactic and citric acid (LCA) have been approved by USDA-FSIS to control food-borne pathogens during industry-scale poultry processing (5). The data available currently on industry-scale poultry processing have reported the efficacy of various commercial antimicrobials to control *Salmonella* and *Campylobacter* in the processing of poultry meat (6, 7). However, few studies have validated the efficacy of commercial antimicrobial interventions on MPPU-produced broiler meat.

Therefore, the present study had two main objectives. First, we wished to ascertain the populations of aerobic plate counts (APCs), total coliforms (TCCs), generic *Escherichia coli*, yeast, and molds on raw broiler carcasses and evaluate the prevalence of *Salmonella* and *Campylobacter* spp. in the ceca and on the carcasses of broilers processed at a university pilot-scale MPPU. Second, we wished to evaluate the efficacy of commercial antimicrobial agents against *Campylobacter jejuni* on MPPU-processed broiler carcasses.

## Materials and Methods

### Raising Broilers

**Figure 1:**
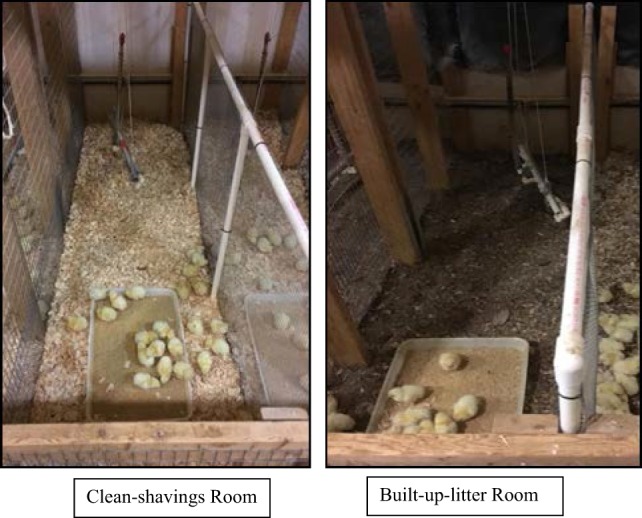
Broilers were reared in “clean-shavings” and “built-up-litter” room.

Broilers sampled for the present study were obtained from a study conducted at the West Virginia University (WVU) Poultry Farm, as reported previously by Glover et al. (8). Broilers were cared for according to guidelines set by the Animal Care and Use Committee of WVU. Briefly, 736 1-day-old straight-run Hubbard × Cobb chickens were obtained from a local hatchery and raised for 38 days with 174 chickens using in this study. Broilers had access to food and water *ad libitum*. Broilers were fed with a high-by product protein diet containing a 30% inclusion of wheat (high in non-starch polysaccharides). The diet formulated did not contain any antibiotics or coccidiostats. Litter was bagged and stored at the end of each replicate (three consecutive identical replicates were conducted) to allow each room to be disinfected appropriately between each replicate. Once rooms had been disinfected, litter and CS were redistributed. Two rooms within the WVU Poultry Farm Research Facility were utilized to completely remove litter and broilers to eliminate cross-contamination. One room was termed “CS” (Figure 1), and the second room was called “BUL” (Figure 1). There were 16 pens in CS or BUL room with 23 birds per pen, and a stocking density was 0.06 m^2^/bird. At the end of each replicate, three to four broilers from eight pens from each room (CS or BUL) were collected and processed at the WVU pilot processing facility that mimicked an MPPU, which was replicated three times with a total of 174 broilers. For each replication, 25 broilers were from CS room, and 24 broilers were from BUL room. Litters from CS and BUL treatment were analyzed for *Salmonella* in a commercial microbial testing lab and no *Salmonella* (<1 CFU/25 g litter) was detected in litters.

### Processing Broilers in an MPPU Facility

The processing of aforementioned broilers was in an MPPU facility at WVU poultry farm with no application of antimicrobial agents. No *Salmonella* spp. was sampled from the MPPU facility according to the real-time polymerase chain reaction (PCR) test of the InvA gene (internal unpublished data). Broilers were killed with a hand knife and allowed for bleeding for 2 min. After scalding and defeathering, the evisceration was conducted manually on a stainless-steel table with glove hands. Broiler carcasses were then rinsed in warm (50°C) tap water before chilling in a static container with ice water for 24 h. Ceca samples of each processed broilers were collected for later microbial analyses.

### Preparation of Broiler Carcasses and Ceca Samples

After chilling for 24 h, carcasses were added to a sterile chicken-sampling bag (Nasco, Fort Atkinson, WI, USA) rinsed with 400 mL of buffered peptone water (BPW; Hardy Diagnostics, Santa Maria, CA, USA) and followed by vigorous shaking for 60 s (9). Ceca samples were prepared by vertical cutting, addition into a sterile filtered Whirl-Pak@ bag (Nasco) with 60 mL of BPW, followed by homogenization in a masticator (IUL Instruments, Barcelona, Spain) for 2 min. The 60 mL of ceca solution was equally split into two tubes for further testing *Salmonella* and *Campylobacter* spp.

### Numeration of APCs, *E. coli*/TCCs, and Yeast/Molds (Y/M)

The rinsate of each carcass sample was serially diluted 10-fold into 0.1% BPW and plated onto APCs, *E. coli*/TCCs, and Y/M petrifilm (3M Microbiology, Saint Paul, MN, USA) for enumeration of the total population of aerobic bacteria, generic *E. coli*, TCCs, and Y/M, respectively, according to manufacturer instructions. Petrifilms were incubated at 25°C for 72 h (APCs), 35°C for 48 h (*E. coli*/TCCs), and 25°C for 120 h (Y/M) followed by manual counting of colonies.

### Isolation of *Salmonella* spp.

The isolation of *Salmonella* spp. was used modified FDA-BAM methods (10) as described in the previous study of Li et al. (11). The aforementioned broiler BPW rinsate and 30 mL of ceca BPW solution were pre-enriched for 24 h at 35°C. Then, 0.1 mL was transferred into a 10 mL of Rappaport–Vassiliadis broth for secondary enrichment (24 h, 35°C). This was followed by streak plating onto XLT-4 agar and HardyCHROM™ agar (Hardy Diagnostics) and incubation for 24 h at 35°C. The one to two presumptive typical *Salmonella* colonies from XLT-4 agar and HardyCHROM agar were confirmed using a *Salmonella* Latex Agglutination Test kit (Oxoid, Basingstoke, UK) and API 20E Test kit (bioMẻrieux, Durham, NC, USA). *Salmonella* Typhimurium ATCC 14028 was used as a positive control from a biochemistry and immunology test.

### Isolation of *Campylobacter* spp.

The isolation of *Campylobacter* spp. was according to the previous study of Scheinberg et al. (9). 30 mL of broiler-carcass rinsate or ceca sample solution was mixed with 30 mL of 2× Bolton’s broth (Hardy Diagnostics). These mixtures were incubated for 48 h at 42°C under microaerophilic conditions (5.0% O_2_, 10% CO_2_, and 85% N_2_) in a 2.5-L microaerophilic jar (Oxoid). Following incubation, a loopful of Bolton’s broth was streaked on modified Campy-Cefex Agar (Hardy Diagnostics) and incubated for 72 h at 42°C under the microaerophilic conditions described above. Presumptive colonies on the modified Campy-Cefex Agar gar were confirmed using the Campy-latex Agglutination Test (Oxoid), oxidase test (Hardy Diagnostics), and Gram staining to observe for “corkscrew” morphology.

### Identification of *C. jejuni* and *Campylobacter coli*

The identified *Campylobacter* colonies were regrown into 10 mL of Bolton’s broth for 48 h at 42°C under the microaerophilic conditions described above. Then, the growing solutions were used to test for the presence of *C. jejuni* and *C. coli* in ceca or carcass samples using a TaqMan^®^ kit (Fisher Scientific, Fair Lawn, NY, USA) following the manufacturer instruction. Total DNA was extracted according to the method described in Li et al. (11) followed by the real-time PCR detection of CadF gene (12). Reactions were conducted in a total volume of 20 µL, which included 10 µL of 2× qPCR MasterMix, 1 µL of *C. jejuni* or *C. coli* primer/probe mix, 1 µL of internal extraction control primer/probe mix, 3 µL of RNAse/DNAse free water, and 5 µL of extracted DNA. Amplification of the CadF gene was done on a 7300 real-time PCR system (Applied Biosystems, Foster City, CA, USA). Conditions for the amplification were 37°C for 15 min, 95°C for 2 min, and 30 cycles of 95°C for 10 s and 60°C for 1 min.

### Preparation of *C. jejuni* Inoculum

Strains RM5032, RM1188, and RM1464 of *C. jejuni* (kindly supplied by Dr. Nereus Gunther from USDA-ARS, Wyndmoor, PA, USA) were used in this study. Each individual *C. jejuni* strain was maintained on Brucella agar (Hardy Diagnostics) at 4°C under microaerophilic conditions (5.0% O_2_, 10% CO_2_, and 85% N_2_) in a 2.5-L microaerophilic jar (Oxoid). The colonies grown on Brucella agar were verified by a Campy-latex Agglutination Test kit. To prepare the inoculum, single colonies of each *C. jejuni* strain were inoculated individually into 10 mL of Bolton’s broth and incubated for 48 h at 42°C under the microaerophilic environment described above. Before experimentation, the three cultures of *C. jejuni* were combined, harvested by centrifugation (5,000 × *g*, 15 min, room temperature), duplicate washed with 0.1% BPW to remove residual media, centrifuged, and resuspended in 0.1% BPW. The bacterial population of the final inoculum suspension was 7 log colony-forming units (CFU)/mL.

### Inoculation of *C. jejuni* on Broiler Carcasses

The MPPU-processed carcasses from WVU poultry farms were transferred to a food microbiology laboratory at WVU and used in experiments within 24–48 h. Broiler carcasses were assigned randomly to a treatment group and inoculated with the three-strain mixture of *C. jejuni*. This was achieved by addition of five drops of 200 µL of the bacterial mixture on medial and lateral sides (13) and placement on foil paper in a biohazard hood for 20 min to allow bacterial attachment. The final inoculation level of the organism on carcasses was 4.54 ± 1.24 log CFU/mL of carcass rinsate.

### Antimicrobial Treatment of Broiler Carcasses

The *C. jejuni*-inoculated broiler carcasses were left untreated (control) or immersed in antimicrobial solutions: PAA (0.1%; pH, 3.0; 15.7°C; Birko, Henderson, CO, USA), LA (5%; pH, 2.0; 15.3°C; Birko), LCA (2.5%; Chicxide^®^), SH (freely available chlorine, 67–69 ppm; pH, 9.1; 14.4°C; Birko), and a PAA/hydrogen peroxide mix (SaniDate^®^ 5.0, 0.25%; pH, 7.25; 15.2°C; Arbico Organics, Tucson, AZ, USA). Treatment involved immersing three carcasses into a 10-L prepared antimicrobial solution with manual agitation (≈500 rpm) for 30 s with draining for 2 min. The tested concentration of PAA, LA, and LCA was in the range allowed in USDA-FSIS Directive 7120.7 (5). PAA concentration was determined using a Titration Drop Test kit (LaMotte Co., Chestertown, MD, USA) (14). The concentration of LA, LCA, and SaniDate 5.0 was calculated according to factsheet supplied by the manufacturer. The initial and residual free-chlorine concentration was measured using the *N*,*N* diethyl-1,4 phenylenediamine sulfate method (15). For SH solution, after 30-s treatment of broiler carcasses, the initial free-chlorine concentration was 67–69 ppm, and the residual free-chlorine concentration was 11.8 ppm. Therefore, the mean initial and final residual free-chlorine concentration was ≈40 ppm (i.e., <50 ppm and in accordance with USDA-FSIS Directive 7120.7) (5). The pH and temperature of antimicrobial solutions were measured using a digital pH meter (Fisher Scientific).

### Microbiological Analyses

Numeration of *C. jejuni* on broiler carcasses was done according to the methods described by Nagel et al. (14) and Gunther et al. (16). Carcasses were placed in a sterile chicken-sampling bag (Nasco) and rinsed with 200 mL of BPW supplemented with 0.1% sodium thiosulfate (Fisher Scientific) followed by vigorous shaking for 60 s (14). After 10-fold serial dilution in Bolton’s broth, the dilution liquid was spread plated onto Brucella agar (16) and incubated for 48 h at 42°C in the microaerophilic jar (5.0% O_2_, 10% CO_2_, and 85% N_2_) before manual counting of colonies. The growth of *Campylobacter* colonies on Brucella agar was also confirmed using the Campy-latex Agglutination test.

### Data Analyses

In study I, three replications were conducted for the experiment. For each replication, treatments of CS (25 broilers) and BUL (24 broilers) were organized in a split-plot design consisting of a 2 × 2 factorial arrangement in a randomized block design for broilers reared at the WVU Poultry Farm. In study II, the antimicrobial intervention test was repeated twice with three carcasses per treatment per repeat (a total of six samples of carcasses per treatment). A chi-square test (significance level at 0.05) from JMP^®^ was done to compare differences in the percentage of *Salmonella, Campylobacter* spp., *C. jejuni*, and *C. coli* on broiler carcasses between treatment of CS and BUL. Data on microbial quality (converted to log CFU/mL) of broiler carcasses (APCs, *E. coli*, TCCs, and Y/M) were analyzed using Student’s *t*-test by SAS v9.2 (SAS Institute, Cary, NC, USA). One-way ANOVA of SAS v9.2 was used to analyze the survival population and reduction of *C. jejuni* on broiler carcasses after antimicrobial treatment. To compare the level of reduction of the *C. jejuni* response to various antimicrobial agents, reduction data were determined using the following equation:

$$\text{Reduction ratio}={\text{log}}_{10}(N_{0}/N)$$

where *N*_0_ is the mean control plate counts and *N* is the plate count of each individual antimicrobial-treated sample. Mean values were compared with a significance level of α = 0.05 as determined by Tukey’s honest significant difference test.

## Results

### Microbial Quality of Broiler Carcasses

As indicators of microbial hygiene, the population of APCs, TCCs, *E. coli*, and Y/M of broiler carcasses from CS and BUL groups is quantified in Table 1. There was no significant difference (*P* > 0.05) in APCs, TCCs, and *E. coli* between carcasses in the CS room and BUL room (Table 1). The mean value (in log CFU/mL) of APCs was 3.4–3.5, TCCs was 2.2–2.5, and *E. coli* was 2.1 of all carcasses (Table 1). The total population of Y/M on CS broiler carcasses was lower by 0.4 log CFU/mL (*P* < 0.05) than BUL carcasses (Table 1).

**Table 1:** Mean ± SD of microbial populations (log CFU/mL of sample rinsate) measured as aerobic plate counts (APCs), total coliforms (TCCs), *Escherichia coli*, and yeast/molds (Y/M) on broiler carcasses in “clean-shavings (CS)” and “built-up-litter (BUL)” rooms.

Treatment	APCs	TCCs	*E. coli*	Y/M
CS (*n* = 75)	3.4 ± 0.2^a^	2.5 ± 0.3^a^	2.1 ± 0.6^a^	1.8 ± 0.3^a^
BUL (*n* = 72)	3.5 ± 0.2^a^	2.2 ± 0.4^a^	2.1 ± 0.5^a^	2.2 ± 0.4^b^

*Mean values with different lowercase letters within a column are significantly different (*P* < 0.05)*.

### Prevalence of *Salmonella* spp. in Broiler Ceca and on Carcasses

The presence of *Salmonella* spp. on broiler carcasses was tested and confirmed by the *Salmonella* Latex Agglutination Test and API 20E strips with a biochemical profile code 6704752 (17). There was no contradiction in results between these two tests. Overall, a *Salmonella* spp. was not detected on any ceca samples tested regardless of CS and BUL treatments, suggesting that *Salmonella* spp. was not colonized in all broilers tested. *Salmonella* spp. was not detected on CS-treated carcasses, and it was present on 2.8% (2 of 72 samples) of carcasses in the BUL room (Table 2).

**Table 2:** Prevalence of *Salmonella* spp. in the ceca and on the carcasses of broilers in “clean-shavings (CS)” and “built-up-litter (BUL)” rooms and processed in a mobile poultry-processing unit.

Treatment	Ceca	Carcasses
CS	0% (0/75)^a^	0% (0/75)^a^
BUL	0% (0/72)^a^	2.8% (2/72)^a^

*Mean values with the same lowercase letters within a column are not significantly different (*P* > 0.05)*.

### Prevalence of *Campylobacter* spp. in Broiler Ceca and on Carcasses

Overall, the prevalence of *Campylobacter* spp. in broiler ceca (64.6–84.6%) and on carcasses (50–56.2%) was shown in Table 3. In general, the prevalence of *Campylobacter* spp. in the ceca in the CS room were lower (*P* < 0.05) than those in the BUL room (Table 3) but similar (*P* > 0.05) on the carcasses of broilers compared to the samples in BUL room (Table 3). Among the broilers in the CS room, *Campylobacter* spp. was colonized in 64.6% (49 of 75) of ceca samples, and was present on 50% (37 of 75) of carcasses (Table 3). Among BUL-treated samples, 84.6% (61 of 72) of ceca samples were colonized with, and 56.3% (41 of 72) of carcasses were carrying *Campylobacter* spp. (Table 3).

**Table 3:** Prevalence of *Campylobacter* spp., *Campylobacter jejuni*, and *Campylobacter coli* in the ceca and on the carcasses of broilers in “clean-shavings (CS)” and “built-up-litter (BUL)” room and processed in a mobile poultry-processing unit.

Treatment	Ceca			Carcass		
	*C*. spp.	*C. jejuni*	*C. coli*	*C*. spp.	*C. jejuni*	*C. coli*
CS	64.6% (49/75)^a^	14.7% (11/75)^a^	36% (27/75)^a^	50% (37/75)^a^	19.4% (14/75)^a^	19.4% (14/75)^a^
BUL	84.6% (61/72)^b^	30.6% (22/72)^b^	30.6% (22/72)^a^	56.3% (41/72)^a^	28.6% (21/72)^a^	25.7% (18/72)^a^

*Mean values with different lowercase letters within a column are significantly different (*P* < 0.05)*.

Quantitative PCR revealed that the prevalence of *C. jejuni* was lower (*P* < 0.05) in the ceca (14.7 vs. 30.6%) but similar on carcasses (19.4 vs. 28.6%) of CS broilers compared to the BUL samples (Table 3). *C. coli* was present at a similar level (*P* > 0.05) in the ceca (36.0 vs. 30.6%) and (*P* > 0.05) on the carcasses (19.4 vs. 25.7%) of CS and BUL-treated samples (Table 3).

### Antimicrobial Efficacy in Inactivation of *C. jejuni*

The survival and reduction values of *C. jejuni* on post-chilled broiler carcasses treated with 0.1% PAA, 5.0% LA, 2.5% LCA, 69 ppm SH, or 0.25% SaniDate 5.0 are shown in Table 4. The initial level of *C. jejuni* recovered on inoculated broiler carcasses was 4.54 log CFU/mL. All tested antimicrobial treatments reduced the *C. jejuni* on broiler carcasses significantly (*P* < 0.05) compared with the untreated control. Specifically, 0.1% PAA reduced *C. jejuni* by 2.04 log CFU/mL compared with the control, which was better (*P* < 0.05) than all the other antimicrobials (Table 4). In the present study, dipping carcasses in 5.0% LA reduced the *C. jejuni* population by 1.43 log CFU/mL compared with the untreated control (*P* < 0.05) (Table 4). Broiler carcasses dipped into SH (69 ppm), 2.5% LCA, and 0.25% SaniDate 5.0 reduced the *C. jejuni* population by 1.65, 1.43, and 1.26 log CFU/mL, respectively, and there were no significant difference (*P* > 0.05) between these treatments (Table 4).

**Table 4:** Survival and reduction (mean ± SD) of *C. jejuni* (counts on Brucella agar) recovered from inoculated broiler carcasses left untreated or treated with peroxyacetic acid (PAA, 0.1%, pH 3.0, 15.7°C), lactic acid (LA, 5%, pH 2.0, 15.3°C), lactic and citric acid (LCA) blend (2.5%, pH 2.7, 15.2°C), sodium hypochlorite (SH, 67–69 ppm, pH 9.1, 14.4°C), a PAA and hydrogen peroxide mixer (SaniDate^®^ 5.0, 0.25%, pH 7.2, 15.2°C) for 30 s.

Treatment	Survival (log CFU/mL)	Reduction (log CFU/mL)
Control	4.54 ± 1.24^a^	–
PAA	2.49 ± 0.77^b^	2.04 ± 0.77^a^
LA	3.11 ± 0.70^b^	1.43 ± 0.70^ab^
LCA	3.11 ± 0.17^b^	1.43 ± 0.71^ab^
SH	2.89 ± 0.15^b^	1.65 ± 0.15^ab^
SaniDate^®^ 5.0	3.28 ± 0.51^b^	1.26 ± 0.51^b^

*“–” indicates reduction data are not available*.

*Mean values with different lowercase letters within a column are significantly different (*P* < 0.05)*.

## Discussion

Aerobic plate counts are used to assess the total microbial population on broiler carcasses. The coliform population (especially the generic *E. coli* population) indicates the potential fecal contamination on processed meat and poultry products according to USDA-FSIS (9). The population of Y/M of processed broiler carcasses has not been reported widely. The value for APCs was similar to, but that for TCCs and *E. coli* was higher than the value noted by Scheinberg et al. (9). They reported that the value (in log CFU/mL) for APCs, TCCs, and *E. coli* was approximately 4.0, 1.5, and 0.9, respectively, in whole chickens at farmers’ markets in Pennsylvania (9). Northcutt et al. (18) found a similar value (in log CFU/mL) for APCs (3.2) and *E. coli* (1.7) on post-chilled conventional chicken rinsates to our results. Although the yeast and molds population recovered from broiler carcasses of CS treatment is significantly lower than the BUL treatment, a 0.4-log difference is generally not considered biologically significant (19). Overall, the levels of APCs, TCCs, *E. coli*, and Y/M found on MPPU-processed broiler carcasses in the present study suggest that small-scale growers of broilers who use MPPUs should implement antimicrobial interventions during processing or apply post-chilling interventions to reduce the background microflora on broiler surfaces.

A high level of *Salmonella* spp. on chickens processed at locally commercialized poultry facility has been reported in other studies (9, 20). For example, Trimble et al. (20) reported that 43% of chicken carcasses processed in an USDA-inspected facility were *Salmonella*-positive. Also Scheinberg et al. (9) found 20–28% of *Salmonella*-positive broiler carcass samples from farmers’ markets and local supermarkets in Pennsylvania. The *Salmonella* present on those small, locally processed broiler carcasses may be attributed to variances in farm management and lack of regulatory guidance. In the present study, very low percentage of *Salmonella* spp. was identified in broiler ceca and on carcasses regardless of CS and BUL treatment, which is in agreement with the studies of Killinger et al. (21) and Trimble et al. (20). They reported that *Salmonella* was not detected on carcasses processed in the university pilot-scale MPPU in the states of Washington (21) and Arkansas (20). These results might be explained by the following four reasons. First, applying good cleaning and sanitization practices could reduce *Salmonella* spp. effectively on broilers (22). The WVU poultry-raising room and pilot MPPU facility was cleaned repeatedly with hot water along with physically sweeping and applying commercial detergent and chlorinated water afterward. Second, compared to the commercial poultry-processing facility, the university pilot-scale MPPU was less frequently used, therefore less cross-contamination would occur. Third, due to budgetary restraints only a limited sample size (23.9%, 174 of 736) of ceca and broiler samples were tested for *Salmonella* spp. Therefore, the results may not accurately reflect the *Salmonella* profile of the entire raised broilers. Finally, in this study, *Salmonella* and *Campylobacter* both occupy the same gastrointestinal tract of broilers; therefore, it is possible that *Campylobacter* was present in significant amounts and *Salmonella* was not detected.

*Campylobacter* spp., especially *C. jejuni* and *C. coli*, are the two major *Campylobacter* species and commonly cause human gastroenteritis if undercooked poultry meat is eaten (12). The percentage of *Campylobacter* spp. on MPPU-processed broilers has not been studied widely. Overall, the prevalence of *Campylobacter* spp. in broiler ceca and on carcasses was much higher than the percentage of *Salmonella*. Findings are in accordance with the study of Trimble et al. (20), which suggest that for small-scale broiler producers, the management practices used to control *Salmonella* effectively might have only a slight effect on *Campylobacter* due to the difference in the physiology and ecology of these two pathogens in production and processing environments (20, 23). The high percentage of *Campylobacter* spp. observed in the present study may have been due to (1) use of a single-stage static scalder; (2) the practice of manual evisceration; (3) a single, static chilling tub without any antimicrobial agents (which may have resulted cross-contamination of broiler carcasses during the pilot-scale MPPU process).

Ceca is the main source of *Campylobacter* colonization in broilers. The impact of CS and BUL on the colonization of *Campylobacter* spp., *C. coli*, and *C. jejuni* in ceca of broilers were investigated in this study. CS reduced the percentage of *Campylobacter* spp. and *C. jejuni* but did not affect the percentage of *C. coli* compared to the BUL treatment. This mixed result might be explained by the two reasons. On one side, BUL was bagged, stored, and maintained the same litter throughout each trial, whereas CS was replaced if “caking” occurred. This process may have allowed for increased colonization of *Campylobacter* spp. and *C. jejuni* in BUL-treated cecum. On the other side, the colonization of *C. coli* might be caused by the complex factors of the broiler cecum, rather than attributed to the practices of litter including using old “dirty” litter repeatedly (24). No significant difference of *Campylobacter* spp., *C. jejuni*, and *C. coli* detected on broiler carcasses regardless of they were reared on BUL or CS treatment indicate that simply application of litter treatment including replacing old dirty litter with CS in the raising room did not directly influence the levels of *Campylobacter* on broiler carcasses. A further antimicrobial intervention is necessary during the post-harvest broiler processing to control *Campylobacter* level in the final broiler carcasses.

Results of this study showed that *Campylobacter* was dominant on MPPU-processed broiler carcasses; therefore, validation of the efficacy of various commercial antimicrobial agents against this pathogen after chilling was important. Nagel et al. (14) and Chen et al. (25) also reported that PAA (0.04–0.1%) is the most effective antimicrobial agent used during post-chilling dipping to decontaminate *Campylobacter* on poultry products compared with chlorine, cetylpyridinium chloride, and lysozyme. Nagel et al. (14) reported that 0.1% PAA achieved a reduction of 2.03 log CFU/mL of *C. jejuni* on broiler carcasses processed in an industry-scale pilot post-chilling dipping tank. Chen et al. (25) found that 0.1% PAA reduced *Campylobacter* by ≈1.5 log CFU/g in ground chicken meat. PAA is a combination of peracetic acid and hydrogen peroxide, and it denatures proteins and disrupts bacterial cell walls (26). PAA at <2,000 ppm (0.2%) has been approved by USDA-FSIS for application on poultry carcasses since 2001 (6, 7), and it is the most prevalent antimicrobial agent used in the poultry industry (7). Small-scale poultry producers in Pennsylvania and the WV area who currently own or will purchase MPPUs wish to know the antimicrobial efficacy of PAA due to the concerns of “organic” processing. The present study provides important, validated data for them.

Lactic acid at <5.0% is approved by the USDA-FSIS as an antimicrobial agent applied on broiler carcasses before or after chilling (5). In the present study, dipping carcasses in 5.0% LA reduced the *C. jejuni* population by 1.43 log CFU/mL compared with the untreated control (*P* < 0.05) (Table 4). Coşansu and Ayhan (27) dipped the legs and breasts of chickens into 1 and 3% LA and achieved reductions of 0.36–1.36 and 1.27–1.98 log MPN/cm^2^, respectively. Burfoot et al. (28) sprayed 4 and 8% LA onto chicken carcasses and reduced the *Campylobacter* on skin surfaces by 0.4–0.8 and 1.9 log CFU/g, respectively. Potential undesirable sensory and quality concerns have been raised upon application of LA on broiler carcasses (29).

In the present study, broiler carcasses dipped into SH (69 ppm), 2.5% LCA and 0.25% SaniDate 5.0 reduced the *C. jejuni* population by 1.65, 1.43, and 1.26 log CFU/mL, respectively, and there were no significant difference (*P* > 0.05) between these treatments (Table 4). Recently, SH (commonly referred to as “free chlorine”) has lost its dominant position as an antimicrobial agent used in poultry-meat processing due to: the requirement of a high concentration; rapid reaction with organic matter; a poultry-meat trade issue between the USA and Russia (7, 14, 25). There is a growing interest in the development and evaluation of other chemical antimicrobials as chlorine alternatives. LCA (Chicxide; a buffered blend of LA and citric acid) at ≤2.5% is approved for use on poultry-meat surfaces (5), and its antimicrobial efficacy against *Salmonella* spp. has been evaluated on broiler carcasses (30). SaniDate 5.0 contains 23% hydrogen peroxide and 5.3% PAA and has been shown to control food-borne pathogens on food-contact surfaces effectively. It is also recommended by the WV Small Farm Center for use on poultry meat for small-scale poultry growers in WV (personal communication with Dr. Tom McConnell, Program Leader of the WV Small Farm Center). Results of the present study suggest a similar reduction effect on *Campylobacter* by LCA and SaniDate 5.0 compared with SH. Hence, LCA and SaniDate 5.0 could be used by local, small-scale MPPU poultry processors during post-chilling.

In conclusion, results of this study suggest that the development of good clean and sanitizing practices may control *Salmonella* on broiler carcasses effectively. Broilers reared on CS could be beneficial for the pre-harvest control of *Campylobacter* compared with broilers reared on BUL. Results of the present study confirmed that application of post-chilling antimicrobial-dipping treatments (especially PAA) could be a potential intervention approach to control *Campylobacter* on locally processed broilers using an MPPU. These results could contribute to the development of the new USDA-FSIS 5-year strategic plan for control of *Salmonella* in poultry-meat products (31). Our data could also assist WV state and local regulatory agencies to assess the potential risk and develop control strategies for *Salmonella* and *Campylobacter* in the application of MPPU processes for local poultry growers.

## Ethics Statement

Broilers sampled for the present study were obtained from a study conducted at the WVU Poultry Farm, as reported previously by Glover et al. (8). Broilers were cared for according to guidelines set by the Animal Care and Use Committee of WVU. Briefly, 736 1-day-old straight-run Hubbard × Cobb chickens were obtained from a local hatchery and raised for 38 days with 174 chickens using in this study. Broilers had access to food and water *ad libitum*. Broilers were fed with a high-by product protein diet containing a 30% inclusion of wheat (high in non-starch polysaccharides). The diet formulated did not contain any antibiotics or coccidiostats. Litter was bagged and stored at the end of each replicate (three consecutive identical replicates were conducted) to allow each room to be disinfected appropriately between each replicate. Once rooms had been disinfected, litter and CS were redistributed. Two rooms within the WVU Poultry Farm Research Facility were utilized to completely remove litter and broilers to eliminate cross-contamination. One room was termed “CS” (Figure 1) and the second room was called “BUL” (Figure 1). There were 16 pens in CS or BUL room with 23 birds per pen, and a stocking density was 0.06 m^2^/bird. At the end of each replicate, three to four broilers from eight pens from each room (CS or BUL) were collected and processed at the WVU pilot processing facility that mimicked an MPPU, which was replicated three times with a total of 174 broilers. For each replication, 25 broilers were from CS room, and 24 broilers were from BUL room. Litters from CS and BUL treatment were analyzed for *Salmonella* in a commercial microbial testing lab, and no *Salmonella* (<1 CFU/25 g litter) was detected in litters.

## Author Contributions

KL conducted poultry processing, microbial pathogen testing, reference organization, and drafted manuscript. LL conducted antimicrobial validation study and data collection and analysis. BG raise all broiler carcasses and revised the manuscript. JM managed the poultry farm and coordinated the project. CS generated the idea of this project and drafted, revised, and finalized the manuscript.

## Conflict of Interest Statement

The authors declare that the research was conducted in the absence of any commercial or financial relationships that could be construed as a potential conflict of interest.
