# Supplementary figures and images for: Impact of Built-up-Litter and Commercial Antimicrobials on Salmonella and Campylobacter Contamination of Broiler Carcasses Processed at a Pilot Mobile Poultry-Processing Unit
Source: Front Vet Sci. 2017 Jun 9;4:88. doi: 10.3389/fvets.2017.00088 (PMC5465233; doi:10.3389/fvets.2017.00088)

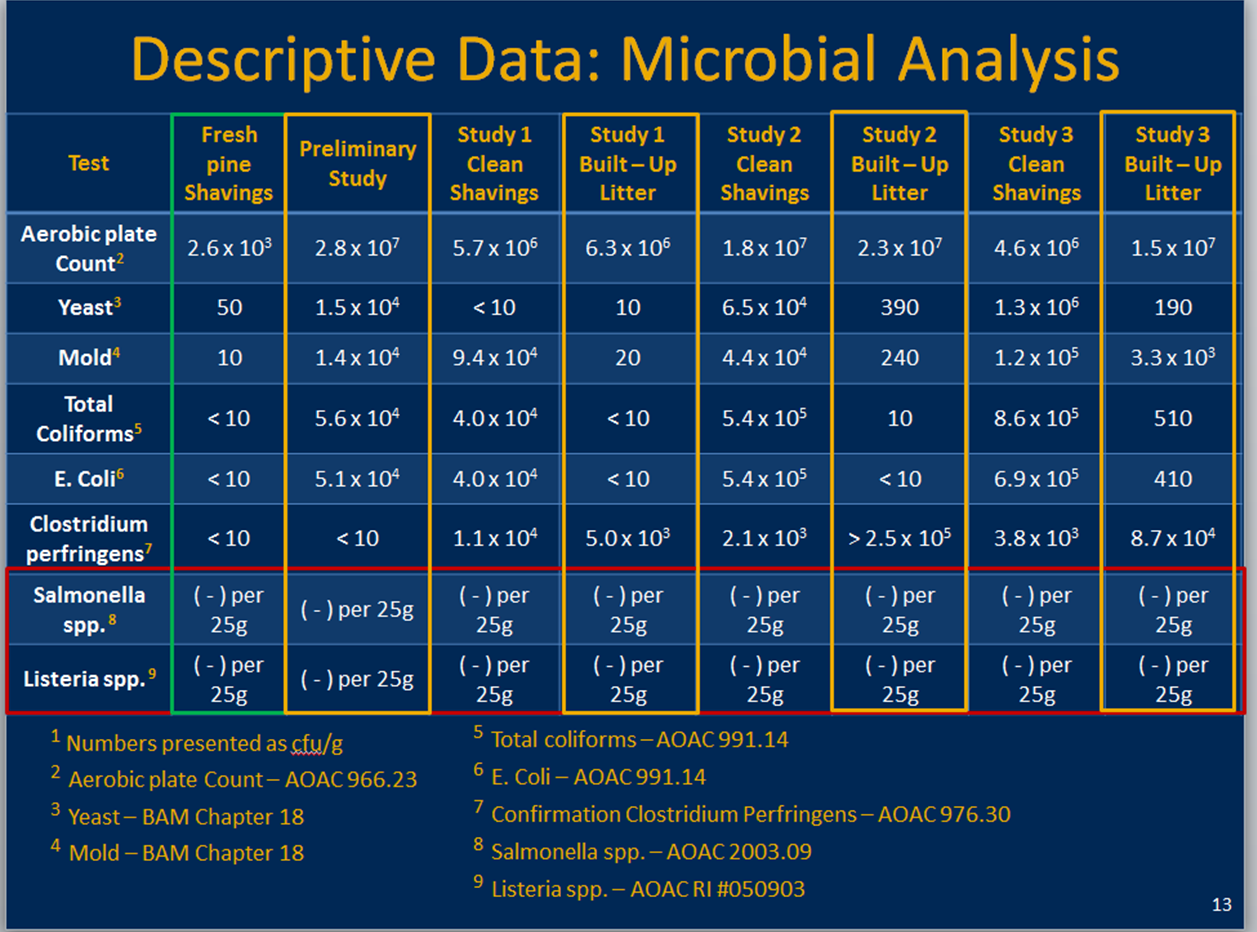

Supplement: Supplementary file 1 [file Table_5.DOCX]
